# Bone Morphogenetic Protein-7 Ameliorates Cerebral Ischemia and Reperfusion Injury via Inhibiting Oxidative Stress and Neuronal Apoptosis

**DOI:** 10.3390/ijms141223441

**Published:** 2013-11-28

**Authors:** Haitao Pei, Dongming Cao, Zhuangli Guo, Guofang Liu, Yunliang Guo, Chenglong Lu

**Affiliations:** Department of Neurology, Affiliated Hospital of Medical College Qingdao University, Qingdao 266003, Shandong, China; E-Mails: peihtao@sohu.com (H.P.); caodongmingdune@163.com (D.C.); gzhl425@163.com (Z.G.); guofangliu1123@sina.com (G.L.); luchenglong2006@sina.com (C.L.)

**Keywords:** bone morphogenetic protein-7, cerebral ischemia/reperfusion injury, oxidative stress, apoptosis

## Abstract

Previous studies have indicated that bone morphogenetic protein-7 (BMP-7) is neuroprotective against cerebral ischemia/reperfusion (IR) injury. The present study was undertaken to determine the molecular mechanisms involved in this effect. Adult male Wistar rats were subjected to 2 h of transient middle cerebral artery occlusion (MCAO), followed by 24 h of reperfusion. BMP-7 (10^−4^ g/kg) or vehicle was infused into rats at the onset of reperfusion via the tail vein. Neurological deficits, infarct volume, histopathological changes, oxidative stress-related biochemical parameters, neuronal apoptosis, and apoptosis-related proteins were assessed. BMP-7 significantly improved neurological and histological deficits, reduced the infarct volume, and decreased apoptotic cells after cerebral ischemia. BMP-7 also markedly enhanced the activities of antioxidant enzymes superoxide dismutase (SOD) and glutathione peroxidase (GSH-PX), and reduced the level of malondialdehyde (MDA) in IR rats. In addition, Western blot analysis indicated that BMP-7 prevented cytochrome c release, inhibited activation of caspase-3, caspase-9 and caspase-8. Our data suggested that BMP-7 has protective effects against cerebral IR injury in rats, and the neuroprotective effects may be attributed to attenuating oxidative stress and inhibiting neuronal apoptosis.

## 1. Introduction

Stroke is a leading cause of death and adult disability worldwide [1,2]. Ischemic stroke accounts for approximately 80% of all strokes, and occurs when a major cerebral artery is blocked by a thrombus or embolism [3]. This blockage leads to brain injury through a complex series of pathophysiological events leading to neuronal cell death and subsequent neurological dysfunction. Thrombolysis with intravenous administration recombinant tissue plasminogen activator (rt-PA) is the only approved treatment for acute stroke, but fewer than 5% of stroke patients are eligible for this therapy, primarily due to the narrow time window (<3 h). Besides, it does not provide reperfusion and it increases the risk of symptomatic intracranial hemorrhage [4]. Thus, there is a compelling need to develop novel therapeutic options for patients with ischemic stroke.

Bone morphogenetic protein-7 (BMP-7), or osteogenic protein-1 (OP-1) is a member of the transforming growth factor (TGF)-β super-family. Both BMP-7 and its receptors (BMPRs) are expressed in the central nervous system [5,6] and can be upregulated after transient global cerebral ischemia [7,8]. Exogenous administration of BMP-7 before methamphetamine (MA) administration reduced the MA-induced increment in cerebral infraction, caspase-3 activation, and behavioral deficits [9]. Pretreatment with BMP-7 prior to general hypoxia or ischemia reduced brain infarction volume and mortality in rats [10,11]. The pre-stroke implantation of fetal kidney cells, a tissue with high levels of BMP-7, into the rodent cortex reduced infarct volume and improved functional recovery [12]. The protective effects of fetal kidney transplantation were attenuated after intracerebroventricular delivery of BMP antagonist noggin [13]. Moreover, post-stroke intracisternal or intracerebroventricular BMP-7 injection partially improves motor function two weeks after ischemia in rats [14,15]. Taken together, these data suggest that exogenously delivered BMP-7 has beneficial effects against ischemia.

*In vitro* studies demonstrated that BMP-7 has anti-oxidant [16] and anti-apoptotic [17] properties. However, the *in vivo* protective effect of BMP-7 on focal cerebral ischemia and its mechanisms have not been well elucidated. Apoptosis is one of the major pathways that leads to cell death after cerebral ischemia/reperfusion (IR) injury [18]. Oxidative stress from reactive oxygen species (ROS) has long been considered as a key deleterious factor in cerebral IR injury and may ultimately result in the initiation of pathways that lead to apoptotic cell death [19]. Considering the facts mentioned above and the role of apoptosis and oxidative stress in the pathophysiology of IR injury, the present study was designed to explore the neuroprotective effects of BMP-7, as well as the underlying mechanisms by focusing on neuronal apoptosis and oxidative stress.

## 2. Results

### 2.1. Neurological Deficits

**Figure 1.**
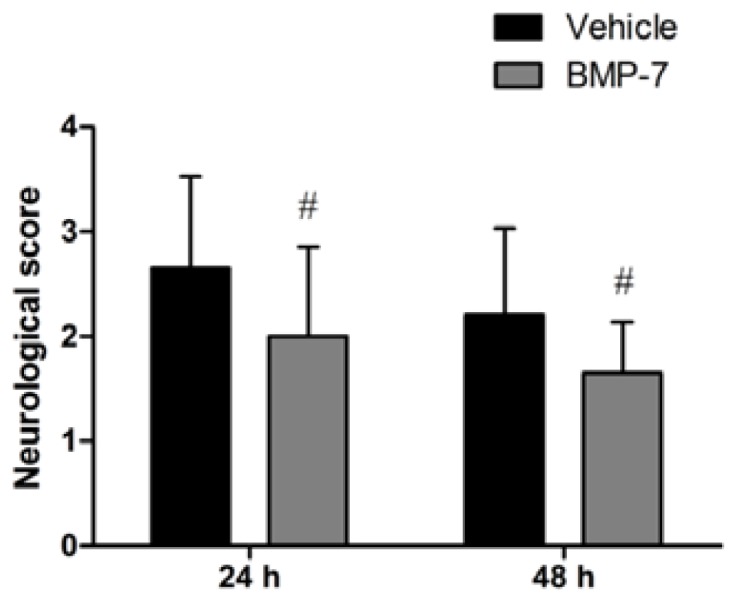
Bone morphogenetic protein-7 (BMP-7) significantly improved neurological recovery compared with the vehicle group at 24 or 48 h after reperfusion. Data were expressed as mean ± SD (*n* = 20); ^#^*p* < 0.05 *versus* vehicle group.

No neurological deficits were seen in the sham group. When tested at 24 or 48 h after reperfusion, vehicle-treated rats displayed severe neurological deficits. BMP-7 treatment significantly improved the neurological score compared with the vehicle group (*p* < 0.05) (Figure 1).

### 2.2. Effects of BMP-7 on Infarct Volume

**Figure 2.**
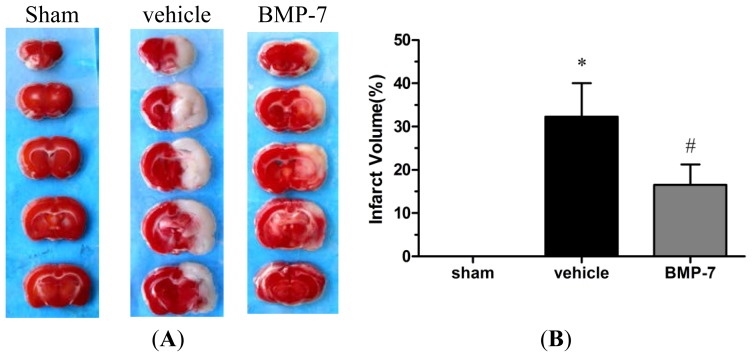
BMP-7 reduces the infarct volume in focal ischemia stroke model. (**A**) Representative pictures of stained cerebral sections in each group. The normal tissue stained dark red, while the infarct tissue was white; (**B**) Quantitative analysis of infarct volume. Data were expressed as mean ± SD (*n* = 10). * *p* < 0.05 *versus* sham group; ^#^*p* < 0.05 *versus* vehicle group.

There was no infarct area observed in the sham group, while in the vehicle group, a significant infarction was observed at 24 h after reperfusion. Under treatment with BMP-7, the infarct volume in the BMP-7 group was significantly smaller than that in the vehicle group (*p* < 0.05) (Figure 2).

### 2.3. Effects of BMP-7 on Histopathology

**Figure 3.**
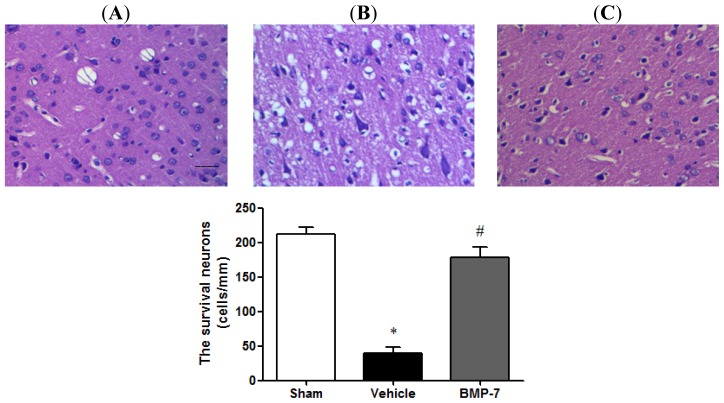
Morphological examinations of cortex neurons by hematoxylin and eosin staining (scale bar = 20 μm). (**A**) sham group; (**B**) vehicle group; (**C**) BMP-7 group. BMP-7 treatment increased the number of surviving neurons and ameliorated the extent of damage in the cortex. Data were expressed as mean ± SD (*n* = 10). * *p* < 0.05 *versus* sham group; ^#^*p* < 0.05 *versus* vehicle group.

To evaluate the effects of BMP-7 on cerebral IR injury, histological examination in each group was performed in HE stained sections. The sham group showed normal neurons with no pathological change. Compared with the sham and BMP-7 group, most of the neurons in the vehicle group had disappeared. In the vehicle group, neuronal loss was severe and dying neurons showed shrunken cytoplasm and pyknotic nuclei. In the BMP-7 group, the number of surviving neurons was markedly increased than that in the vehicle group (*p* < 0.05), and the extent of damage was significantly ameliorated as well (Figure 3).

### 2.4. Effects of BMP-7 on MDA Content and Antioxidant Enzymes Activities

**Figure 4.**
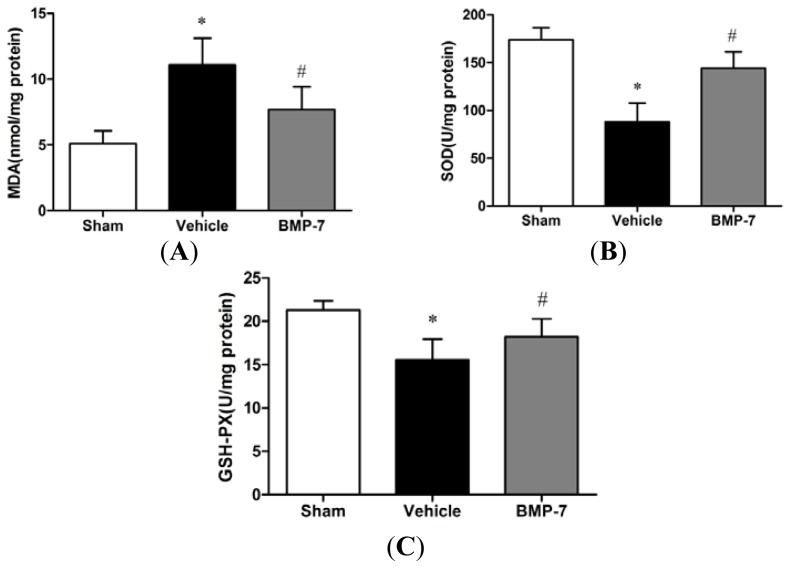
BMP-7 ameliorates oxidative stress after cerebral ischemia/reperfusion (IR) injury. BMP-7 significantly decrease the content of malondialdehyde (MDA) (**A**); and enhanced superoxide dismutase (SOD) (**B**); and glutathione peroxidase (GSH-PX) (**C**) activities in ischemia reperfusion rats. Data were expressed as mean ± SD (*n* = 10). * *p* < 0.05 *versus* sham group; ^#^*p* < 0.05 *versus* vehicle group.

To investigate whether BMP-7 affects oxidative stress damage, we evaluated the content of MDA and the activities of antioxidant enzyme in the penumbral cortex. The content of MDA is an index of lipid peroxidation, which increased significantly after cerebral IR injury (*p* < 0.05). BMP-7 treatment significantly decreased the MDA content compared with the vehicle group (*p* < 0.05). Additionally, the activities of antioxidant enzymes including SOD and GSH-PX were decreased significantly in the vehicle group as compared with the sham group (*p* < 0.05). However, BMP-7 treatment induced significant elevation of SOD and GSH-PX activities compared with the vehicle group (*p* < 0.05) (Figure 4).

### 2.5. Effects of BMP-7 on Neuronal Apoptosis

**Figure 5.**
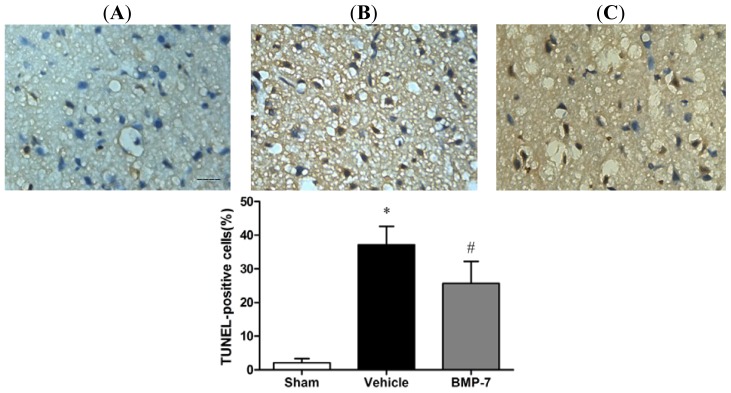
BMP-7 protect neuronal cell against cerebral ischemia-induced apoptosis. The TUNEL-positive apoptotic cells were labeled brown in nuclei (scale bar = 20 μm). (**A**) sham group; (**B**) vehicle group; and (**C**) BMP-7 group; Apoptotic rates significant increased at 24 h after reperfusion in the vehicle group. BMP-7 treatment could decrease apoptotic rate. The apoptotic rate was calculated as percentage of apoptotic cells among total nerve cells, and data were expressed as mean ± SD (*n* = 10). * *p* < 0.05 *versus* sham group; ^#^*p* < 0.05 *versus* vehicle group.

Neuronal injury in the penumbral cortex was analyzed by TUNEL staining. TUNEL-positive apoptotic cells were sparsely detected in the sham group; while the apoptotic rate was significantly increased in the vehicle group compared with the sham group (*p* < 0.05). BMP-7 treatment effectively attenuated the neuronal death caused by cerebral IR injury, as indicated by significant reduction of apoptotic rate (*p* < 0.05) (Figure 5).

### 2.6. Effects of BMP-7 on Cytochrome C Release, Cleaved Caspase-3, Cleaved Caspase-9 and Cleaved Caspase-8 Expression after Cerebral IR Injury

**Figure 6.**
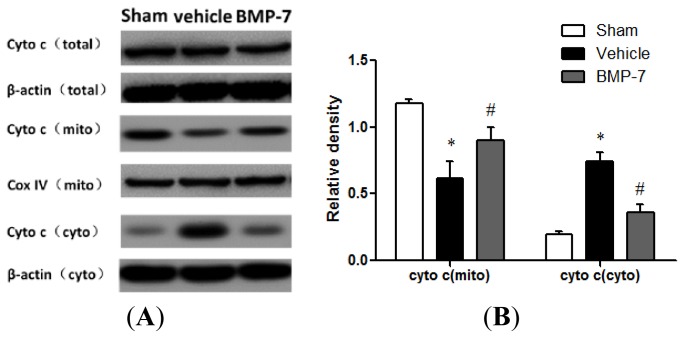
BMP-7 inhibits the release of cytochrome c release from mitochondria to cytosol in cerebral IR injury rats. (**A**) Immunoblots of total cytochrome c, cytochrome c from mitochondrial fraction, and cytochrome c from cytosolic fraction after 24 h of reperfusion; and (**B**) Quantitative analysis of cytochrome c protein levels in the mitochondrial fraction and cytosolic fraction; COX IV served as equal mitochondria loading control and β-actin served as equal cytosol loading control. Data were expressed as mean ± SD (*n* = 10). * *p* < 0.05 *versus* sham group; ^#^*p* < 0.05 *versus* vehicle group.

We evaluate cytochrome c release, cleaved caspase-3, cleaved caspase-9 and cleaved caspase-8 expression, to try to elucidate possible neuroprotective mechanisms of BMP-7. Western blotting showed that there are no significant differences in total cytochrome c expression among the three groups. Compared with the sham group, the cytochrome c expression increased significantly in the cytosolic fraction but decreased in the mitochondrial fraction after 24 h of reperfusion in the vehicle group (*p* < 0.05). BMP-7 treatment markedly elevated the expression of cytochrome c in mitochondrial fraction and reduced that in the cytosolic fraction (*p* < 0.05) (Figure 6). These results suggested that BMP-7 could significantly inhibit the release of cytochrome c.

**Figure 7.**
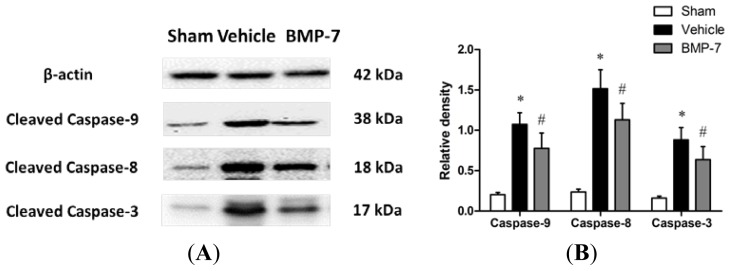
Protein expression of cleaved caspase-3, cleaved caspase-9 and cleaved caspase-8 in the penumbral cortex was detected by Western blotting analysis. (**A**) Representative Western blots of cleaved caspase-3, cleaved caspase-9, cleaved caspase-8 and β-actin; and (**B**) Quantitative analysis of target protein levels in each group. Data were expressed as mean ± SD (*n* = 10). * *p* < 0.05 *versus* sham group; ^#^*p* < 0.05 *versus* vehicle group.

The expression of cleaved caspase-3, cleaved caspase-9 and cleaved caspase-8 were detected in different groups for further investigation. From the Western blot analysis, cleaved caspase-3, cleaved caspase-9 and cleaved caspase-8 were upregulated after cerebral IR injury; however, these changes were remarkably attenuated by BMP-7 treatment (*p* < 0.05) (Figure 7).

## 3. Discussion

BMP-7 is a member of BMPs subfamily that has already been approved by Health Canada and the Food and Drug Administration (FDA) and used to treat acute fractures and spinal fusions in more than 340,000 patients worldwide [20]. Apart from its bone formation effect, BMP-7 also exhibits powerful effects against IR injury in a wide range of organs including brain [9], kidney [21], liver [22], and heart [23].

Previous experiments have demonstrated that rats intravenously receiving BMP-7 at a dose of 10^−4^ g/kg had significantly enhanced locomotor activity, however, these behavioral parameters were not significantly enhanced in rats receiving a higher dose of BMP-7 (10^−3^, 10^−2^ g/kg) [8]. Moreover, BMP-7 has a short life (about 10–30 min) and works best within a short range. Thus, we selected 10^−4^ g/kg as the effective dosage of BMP-7, and administered it intravenously at the onset of reperfusion. In this study we demonstrated that BMP-7 protected against cerebral IR injury by reducing infarct volume, improving neurological and histological deficits, and these beneficial effects were associated with inhibition of oxidative stress or neuronal apoptosis-related pathways, such as reduction of the MDA content, elevation of SOD and GSH-PX activities, inhibition of cytochrome c release, and suppression of caspase-3, caspase-9 and caspase-8 activation.

Under physiological conditions ROS are generated at low levels and play important roles in signaling and metabolic pathways [24], however, under pathologic conditions such as IR, their overproduction lead to oxidative stress, causing cell damage to nervous tissue, which may lead to DNA oxidation, promoting chain reactions of membrane lipid peroxidation, and alterations in membrane fluidity [19,25,26]. ROS produces malondialdehyde (MDA), an end product of lipid peroxidation. Therefore in the present study the level of MDA was estimated to estimate extent of ROS. Our result showed that the elevated level of MDA was markedly decreased by treatment with BMP-7, indicating that the neuroprotection conferred by BMP-7 may be attributed to attenuating lipid peroxidation following transient global cerebral ischemia. The overproduction of ROS can be detoxified by endogenous antioxidants, causing their cellular stores to be depleted [27]. Superoxide dismutase (SOD) and glutathione peroxidase (GSH-PX) are thought to be two dominant enzymes acting as free radical scavengers that could prevent the deleterious stroke-induced ROS generation [28]. SOD scavenges the superoxide anion radical (O^−^_2_) by catalyzing its dismutation to H_2_O_2_, which is scavenged to water by GSH-PX at the expense of glutathione [26]. In the present study, BMP-7 was found to be effective in stimulating the activities of SOD and GSH-PX. Previous studies have demonstrated that BMP-7 attenuates hypoxia-induced or H_2_O_2_-induced increase in LDH activity in primary cortical cultures [16]. These data suggest that BMP-7 afford protection against cerebral IR injury through the amelioration of oxidative stress.

Apoptosis plays a significant role in regulating cell death after cerebral IR injury [18]. Oxygen deficit due to ischemia may exhaust the reserved ATP and lead to mitochondrial energy abnormality, which further initiates mitochondria dysfunction and the activation of apoptotic mediators [29,30]. In this study, a great number of apoptotic cells were found in the penumbral cortex of vehicle group at 24 h after reperfusion, whereas BMP-7 treatment reduced neuronal apoptosis. These results are consistent with recent findings showing that the neuroprotection of BMP-7 is probably through inhibition of apoptosis [31,32].

Mitochondria play a pivotal role in regulating apoptotic pathways by releasing pro-apoptotic proteins such as cytochrome c, apoptosis inducing factor, and Smac/DIABLO [33]. Accumulating evidence has shown that the release of cytochrome c from mitochondria intermembrane space to the cytoplasm triggers a cascade of pathological processes that lead to neuronal apoptosis [34,35]. There are no significant differences in total cytochrome c expression among the three groups, however, we found that a remarkable release of cytochrome c from mitochondria to cytosol at 24 h after reperfusion, which was alleviated significantly by treatment with BMP-7. Further investigation showed suppressed activation of caspase-3, caspase-9 and caspase-8 in the BMP-7 treated group. Caspase-9 is a crucial up-stream initiator enzyme in the mitochondria-mediated apoptotic pathway and its activation can be achieved either by cleavage or by association with apoptotic protease activating factor 1 (Apaf-1) to form the active apoptosome [36]. In the death receptor pathway, ligation of a receptor belonging to the tumor necrosis factor (TNF) receptor family leads to the induction of apoptosis through activation of caspase-8 [37]. Both activated caspase-9 and activated caspase-8 can further catalyze down-stream caspases including the major effector enzyme caspase-3, which leads to DNA fragmentation and subsequently to apoptotic cell death. In our experiments, the increased activation of caspase-3, caspase-9 and caspase-8 reaches the high level at 24 h after reperfusion, which could be effectively attenuated by BMP-7 treatment. Recent evidence suggests that exogenous administration of BMP-7 reduces the MA-induced increment in caspase-3 activation [9]. These findings suggest that the neuroprotection of BMP-7 is through inhibition of both intrinsic (mitochondrial) pathway and extrinsic (death receptor) pathway.

## 4. Materials and Methods

### 4.1. Animals

Male Wistar rats weighing 250–270 g were obtained from the Experiment Animal Center of Qingdao Drug Inspection Institute (SCXK (LU) 20090007). All animals were housed in polypropylene cages at an ambient temperature of 22 ± 2 °C, relative humidity 60% ± 10% under 12 h light/dark cycle in an animal house. They were allowed free access to food and water before surgery. The study protocol was approved by the Ethics Committee of Qingdao University.

### 4.2. Experimental Groups

Rats were randomly divided into three groups: (1) sham group; (2) vehicle group, IR injury with phosphate-buffered saline vehicle injected into tail vein at the onset of reperfusion; (3) BMP-7 group, IR injury with BMP-7 (10^−4^ g/kg) injected into tail vein at the onset of reperfusion.

### 4.3. Induction of Cerebral IR Injury

The cerebral IR injury model in rat was similar to that described previously [38]. Briefly, rats were anesthetized with 10% chloral hydrate (400 mg/kg, intraperitoneally). A 4.0 monofilament nylon suture with a tip rounded tip was introduced into the right external carotid artery (ECA) and gently advanced approximately 17–18 mm from the bifurcation of the common carotid artery (CCA) to occlude the middle cerebral artery. After 2 h of ischemia, the filament was carefully removed to generate reperfusion injury. Rectal temperature was maintained at 36.5–37.5 °C throughout the surgery using an incandescent lamp. Sham-operated rats underwent the same surgical procedures without inserting a filament.

### 4.4. Neurological Evaluation

Neurological deficits were evaluated by an observer blinded to the treatment of animals after 24 and 48 h of reperfusion according to the method previous described [38]. Score 0: no neurologic deficit; Score 1: failure to extend left forepaw fully; Score 2: circling to the left; Score 3: falling to the left; Score 4: did not walk spontaneously and had a depressed level of consciousness. Rats that did not show neurological deficits immediately after reperfusion (neurological score = 0) were excluded from the groups.

### 4.5. Measurement of Infarct Volume

At 24 h after reperfusion, rats were anesthetized and then sacrificed (*n* = 10 in each group). The brains were quickly removed and sliced into 5 coronal sections 2-mm thick. Each slice was immersed in 2% TTC saline solution and incubated at 37.5 °C for 15 min, followed by 10% formalin fixation overnight. The infarct areas and the hemisphere areas of each slice were determined by an image analysis system (Image-pro plus 6.0, Media Cybernetics, Bethesda, MD, USA). The infarct areas on each slice were summed and multiplied by slice thickness to give the infarct volume. To compensate for edema and atrophy, the corrected volume was calculated using the following equation: Percentage hemisphere lesion volume = ((total infarct volume − (right hemisphere volume − left hemisphere volume))/left hemisphere volume) × 100%.

### 4.6. Histological Assessment

Twenty-four hours after reperfusion, rats (*n* = 10 in each group) were anesthetized and transcardially perfused with saline for 5 min, following by 4% paraformaldehyde (PFA) in phosphate-buffered saline. The brains were post fixed in 4% PFA overnight and then embedded with paraffin. A tissue block spanning from the optic chiasm to 4 mm posterior to it was cut and sliced into 5 μm sections until use. These paraffin blocks were also used for TUNEL staining. Hematoxylin and eosin staining was performed to assess the histological injury. The damage was evaluated by counting the number of surviving neurons per 1 mm of cortex cells under a light microscope.

### 4.7. TUNEL Staining

TUNEL staining was performed to visualize DNA fragmentation using a TUNEL Apoptosis Detection Kit (Jiancheng Institute of Bioengineering, Nanjing, China). Briefly, sections were digested by 20 μg/mL proteinase K for 15 min at 37 °C, incubated with TUNEL reaction mixture at 37 °C for 60 min and transferred to wash buffer for 30 min. Finally, the sections were visualized using a converter-POD with 0.05% 3,3-diaminobenzidine (DAB). TUNEL-positive cells in the penumbral cortex were semi-quantitatively scored in five fields.

### 4.8. SOD, GSH-PX Activity, and MDA Content Measurement

The rats (*n* = 10 in each group) were anesthetized and decapitated at 24 h after reperfusion. Cortical tissue from the penumbral cortex was immediately isolated and stored at −80 °C until use. Cortical tissue was homogenized in ice cold phosphate buffer to make a 10% homogenate. Then the homogenate was centrifuged at 3000 rpm for 15 min. Superoxide dismutase (SOD), glutathione peroxidase (GSH-PX), and malondialdehyde (MDA) in the supernatant were measured by using commercially available kits (Jiancheng Institute of Bioengineering, Nanjing, China). All assays were conducted according to the manufacturer’s instructions.

### 4.9. Preparation of Mitochondrial and Cytosolic Fraction

Cortical tissues were homogenized in ice-cold isolation buffer. The nuclei and unbroken tissue were sedimented by centrifugation at 1000× *g* for 5 min. The supernatant was subjected to further centrifugation at 12,000× *g* for 10 min. The resulting pellet, which consisted of mitochondrial fraction, was gently re-suspended in isolation medium. The supernatant was then centrifuged at 12,000× *g* for 10 min to obtain the cytosolic fraction. All procedures were performed at 4 °C. The protein content of each sample was measured by Bicinchoninic Acid (BCA) protein assay.

### 4.10. Western Blot Analysis

Proteins extracted from the cytosolic and mitochondrial fraction were collected for detecting the release of cytochrome c. Cortical tissues for cleaved caspase-3, cleaved caspase-9 and cleaved caspase-8 assay were isolated and homogenized in ice-cold lysis buffer. Proteins were extracted and protein concentrations were then determined by BCA protein assay. Thirty micrograms of protein from each sample were separated by SDS-polyacrylamide gels and transferred onto polyvinylidene fluoride membranes. The membranes were blocked with 5% nonfat dry milk in Tris-buffered saline at room temperature for 60 min. The primary antibodies were added and incubated at 4 °C overnight including anti-cleaved caspase-3 (1:1000; Cell Signaling Technology, Boston, MA, USA), anti-cleaved caspase-9 (1:1000; Cell Signaling Technology, Boston, MA, USA), anti-cleaved caspase-8 (1:1000; Cell Signaling Technology, Boston, MA, USA), or anti-cytochrome c (1:1000; Santa Cruz, CA, USA), followed by one hour incubation with peroxidase-conjugated secondary antibodies at room temperature. The protein bands were visualized using an enhanced chemiluminescence detection kit (Amersham Biosciences, Piscataway, NJ, USA). The signals were quantified by scanning densitometry using a Bio-Image Analysis System (Bio-Rad, Hercules, Richmond, CA, USA), and β-actin served as internal control.

### 4.11. Statistical Analyses

All date were expressed as mean ± SD. Statistical analysis of data was performed using one-way ANOVA followed by the LSD post hoc test. A difference was considered significant at *p* < 0.05.

## 5. Conclusions

In summary, our data indicate that BMP-7 has protective effects against cerebral IR injury in rats, and the neuroprotective effects may be attributed to attenuating oxidative stress and inhibiting neuronal apoptosis. The findings provide further insight into the mechanism by which BMP-7 exerts its neuroprotection and suggest that BMP-7 might be of therapeutic value for the treatment of ischemic stroke.
